# Effects of Different Resistance Exercise Forms on Body Composition and Muscle Strength in Overweight and/or Obese Individuals: A Systematic Review and Meta-Analysis

**DOI:** 10.3389/fphys.2021.791999

**Published:** 2022-02-18

**Authors:** Xinhong Liu, Ye Gao, Jiandong Lu, Qirui Ma, Yajun Shi, Jingqi Liu, Shuai Xin, Hao Su

**Affiliations:** ^1^The School of Sports Science, Beijing Sport University, Beijing, China; ^2^School of Physical Education, Northeast Normal University, Jilin, China

**Keywords:** resistance exercise, body composition, overweight and obesity, muscle strength, forms

## Abstract

**Purpose:**

This study is a systematic review and meta-analysis to determine the effects of different forms of resistance training on body composition and muscle strength in overweight and/or obese people.

**Method:**

Only randomized controlled trials (RCTs) were included by searching relevant databases such as a web of science, PubMed, and EBSCO, with search dates up to September 30, 2021. These trials performed resistance exercise training in overweight and/or obese people, and outcome indicators included evaluation of body composition and muscle strength, among other relevant indicators. The Cochrane evaluation tool was used to evaluate the methodological quality of the included literature, and statistical analysis was performed using the R analysis software.

**Results:**

Fifteen studies, 18 trials, with a total of 669 participants meeting eligibility criteria were included in the final analysis, which included three resistance training types (own body weight, resistance bands, and free weight). The results showed that resistance bands improved body fat (BF; SMD −0.79, 95% CI −1.25 to −0.33, *I*^2^ = 0%) in overweight or obese people better than other resistance training types. Own body weight resistance training was better for increasing skeletal muscle mass in overweight or obese people (SMD 0.48, 95% CI 0.04–0.92, *I*^2^ = 0%). In addition, for muscle strength increase, although resistance exercise was shown to improve muscle strength, there was no significant difference between the three exercise forms compared.

**Conclusion:**

Resistance bands can improve body composition by reducing BF. Resistance bands can improve body composition by reducing BF, while it is more effective in increasing muscle mass and own body weight. Therefore, for overweight and obese people, resistance bands resistance exercise can be taken for fat loss, and resistance exercise for own body weight for further muscle gain and maintenance of muscle mass, so as to achieve the purpose of improving body composition.

## Introduction

Nowadays, obesity has become one of the most important factors that endanger people's health. Especially in Europe and the United States (Flegal et al., 2016), nearly one-third of the population is diagnosed as overweight or obese (Olds et al., 2010). Obesity can lead to a range of health problems, including the induction of cardiovascular diseases (Barrett-Connor, 1985; Aslibekyan and Garvey, 2017), insulin resistance (Cortés et al., 2020), and psychological disorders (Erermis et al., 2004). The most direct of these is that it causes a decrease in skeletal muscle contractile function (Bollinger, 2017; Tallis et al., 2018), which leads to a decrease in skeletal muscle strength. Relevant studies have also shown that the relative muscle strength of overweight and obese people is significantly lower than that of healthy people (Bollinger, 2017), which can lead to decreased physical function and even the occurrence of lifelong disability (Choi et al., 2016; Eshima et al., 2017). Therefore, improving body composition and increasing muscle strength in overweight and obese populations has become an urgent issue to be addressed.

Usually, overweight and/or obese people adopt aerobic exercise to reduce fat mass and, thus, improve body composition. However, often aerobic exercise reduces fat mass with a significant decrease in muscle mass and muscle strength (Villareal et al., 2017). Resistance training, as a representative of anaerobic training, can increase muscle strength by improving muscle performance and, thus, muscle strength. Moreover, the resistance exercise not only leads to a decrease in fat mass but also increases lean body mass, thus potentially making a person stronger in absolute terms (Shaibi et al., 2006; McGuigan et al., 2009). However, the effects of different resistance training forms may differ, and their effects on muscle strength and muscle mass are unclear. Additionally, the improvement of body composition by resistance training is still unknown. Therefore, it is particularly important to explore the improvements in body composition and muscle strength with different resistance exercise forms.

Although several meta-analyses have summarized the effects of resistance training on muscle gain and fat loss (Orange et al., 2020), the studies have not specifically investigated which resistance exercise forms are more effective in improving body composition and muscle strength, especially in overweight and obese people. Therefore, the purpose of this meta-analysis was to systematically review and meta-analyze the effects of different forms of resistance training on body composition and muscle strength in overweight and/or obese populations. Additionally, to investigate suitable resistance exercise forms for overweight and obese populations to inform future personalized exercise prescriptions.

## Method

### Search Strategy

Databases, such as Web of Science, PubMed, EBSCO, CNKI, Wanfang, and Weipu, were searched through September 30, 2021. Search terms included “resistance training,” “strength training,” “resistance exercise,” “weight training,” “overweight,” “obesity,” “Body Weight,” “strength,” “power,” and “Body Compositions.” Use the standard Boolean operators (AND, OR) to concatenate search terms, e.g., PubMed's search formula is as follows: ((resistance training [Text Word]) OR (strength training [Text Word])OR (resistance exercise [Text Word])OR (weight training [Text Word])) AND ((overweight [Text Word]) OR (obesity [Text Word]) OR (Body Weight [Text Word])) AND ((strength [Text Word]) OR (power [Text Word]) OR (Body Compositions [Text Word])), and track references for inclusion in the literature and related reviews.

### Inclusion Criteria

Inclusion criteria followed the PICOS principles (i.e., population, intervention, comparison, outcome, and study design): (1) all populations included in this study were overweight and/or obese; (2) the intervention needed to include at least 4 weeks of exercise intervention and had to be resistance training; (3) the control group remained in the same condition as the study group, but without the exercise intervention; (4) outcomes needed to include: body mass index (BMI), body fat (BF), fat-free mass (FFM), skeletal muscle content (SMC), and muscle strength (MS); (5) the type of study was a randomized controlled trial (RCT); and (6) the mean and SD were reported in the trial.

### Exclusion Criteria

Trials that met the following exclusion criteria were excluded: (1) meeting abstracts, case reports, reviews, overviews, and experimental animal studies; (2) the results did not meet the inclusion criteria; (3) study type was not an RCT; and (4) mean and SD could not be obtained from the article or authors.

### Studies Selection

Two reviewers (XL and JL) independently reviewed the titles or abstracts of all studies. A thorough and careful review of relevant studies was conducted to assess whether they could be included in the studies reviewed. Any disagreements were resolved by discussion or consultation with 89 a third author (YG) if necessary.

### Quality Assessment

The Cochrane Collaboration tool was used to assess the risk of bias in the included trials. Two reviewers (QR and YJ) independently assessed the following seven areas of bias: random sequence generation (selection bias), allocation concealment (selection bias), blinding of participants and personnel (performance bias), blinding of outcome assessment (detection bias), incomplete outcome data (attrition bias), and selective reporting (reporting bias). Three levels of high, low, or unclear bias were indicated for each study. Disagreements were resolved by discussion or consultation with a third independent reviewer (XS), if necessary.

### Data Extraction

Two reviewers independently extracted the following data from each included eligible trial: study characteristics (i.e., author and year), participant characteristics (i.e., age and number of participants), and description and outcome of the intervention. Any disagreements were resolved through discussion to reach an agreement, and the authors of the trials were contacted directly to obtain the original studies and data when necessary.

### Statistical Analysis

All statistical analyses were performed using R (RStudio V4.19, Boston, MA, USA) and meta-packages. The percentage change between studies indicating heterogeneity was reported using the *I*^2^ statistic and the chi-square test. Interpretation of the *I*^2^ statistic was by the Cochrane guidelines as follows: low heterogeneity is assumed when *I*^2^ <25%, moderate heterogeneity is assumed when *I*^2^ <75% and >25%, and high heterogeneity is assumed when *I*^2^ > 75% (Higgins et al., 2003). Evidence of heterogeneity is indicated by statistically significant effects based on the chi-square test. If *p* < 0.05, it is considered as a significant difference. Sensitivity analysis was used to investigate the sources of heterogeneity and to assess the stability of the results by removing each test individually. All experimental data were continuous variables. The value of the quantitative data was expressed by the mean difference (MD) and 95% CI. If more than 9 trials were included, possible publication bias was assessed by the funnel plot asymmetry and Egger's test.

## Results

### Search Results

**Figure 1:**
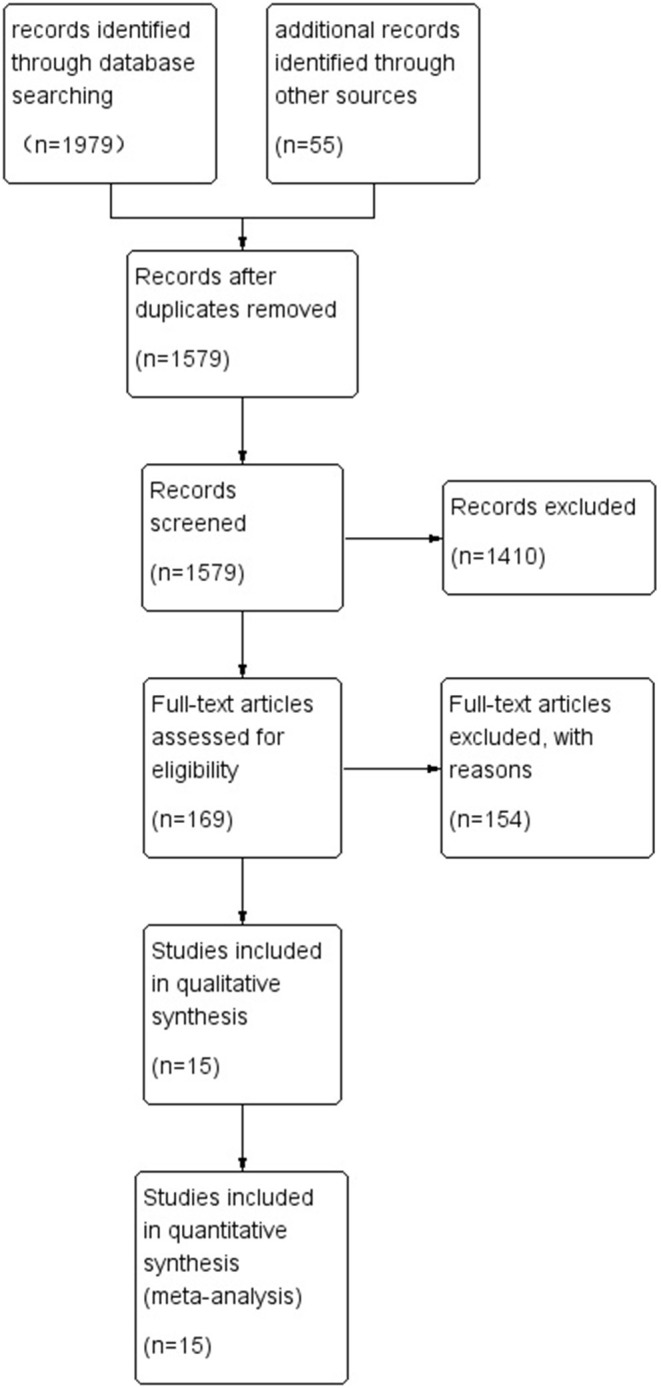
Flow diagram of the study selection.

A total of 1,979 studies were retrieved in the database according to the search strategy, and an additional 55 studies were included through other sources (e.g., personal communication with academic peers and searching reference lists). A total of 455 duplicates were removed from the Endnote literature management software, 1,410 studies were excluded after reading the titles and abstracts, 154 studies were excluded after reading the full text and eligibility assessment, and 15 studies were finally included (Avila et al., 2010; Hernán Jiménez and Ramírez-Vélez, 2011; Cardoso et al., 2013; Lee and Kuk, 2013; Schranz et al., 2014; Benito et al., 2015; Chen et al., 2017; Hassannejad et al., 2017; Huang et al., 2017; Liao et al., 2017; Cunha et al., 2018; Fritz et al., 2018; Hintze et al., 2018; Reljic et al., 2021; Rojo-Tirado et al., 2021), and the literature screening process is shown in Figure 1.

### Description of Included Studies

The characteristics of the included studies are described in Table 1, including first author name, year of publication, age, sex, sample size, intervention methods, and outcomes. A total of 15 of the included studies were published in 2010 and later, and a total of 669 subjects were included who met the BMI values for overweight and obesity in the country. Six of the studies had resistance forms as own body weight, five studies had free weight, and four studies had resistance bands.

**Table 1:** Characteristics of included studies.

**Froms**	**Study**	**Age**	**Sex**	**n**	**BMI**	**Group**	**Duration (week)**	**Frequency (n/week)**	**Dietary intervention**	**Intensity**	**Outcomes**
Own body weight	Lee and Kuk, 2013	12–18	M	14	≥95th percentile	RT	12	3	55–60% carbohydrate, 15–20% protein, and 20–25% fat	No mention	BF; FFM
				11		CON					
Own body weight	Hernán Jiménez and Ramírez-Vélez, 2011	18–35	M and F	8	29.1 ± 2.8	RT	8	4	No	50% 1RM	BMI
				8	30.1 ± 1.9	CON					
Own body weight	Benito et al., 2015	18–50	M and F	24	33.28 ± 1.81	RT	22	3	Hypocaloric diets (between 5,028 and 12,570 kJ)	15RM	BMI; BF; FFM
				22	3.11 ± 2.74	CON					
Own body weight	Cardoso et al., 2013	20–40	F	12	31.8 ± 1.9	RT	4	3	20 g green tea per day	70%RM	BMI; BF; FFM
				12	31.1 ± 1.4	CON					
Own body weight	Hintze et al., 2018	No mention	F	25	32.1 ± 3.8	RT	52	3	55, 30, and 15% of energy intake from carbohydrates, fats, and proteins	70–80%RM	BMI; BF; FFM
				29	31.6 ± 4.2	CON					
Own body weight	Cunha et al., 2018	≥60	F	21	27.1 ± 4.3	RT	12	3	No	No mention	BF; SMC; MS
				20	26.7 ± 4.8	RT 3					
				21	26.7 ± 4.6	CON					
Free weights	Schranz et al., 2013	13–17	M and F	30	31.8 ± 3.7	RT	26	3	No	No mention	BMI; BF; FFM; MS
				26	32.3 ± 4.8	CON					
Free weights	Rojo-Tirado et al., 2021	18–50	M and F	43	31.6 ± 0.5	RT	22	3	Hypocaloric diets (29–34% from fat, 50–55% from carbohydrates, and 20% from protein)	15RM	BMI; BF; FFM
				40	30.7 ± 0.5	CON					
Free weights	Avila et al., 2010	60–75	M and F	12	31.6 ± 3.8	RT	10	3	Modified DASH diet for weight loss	No mention	MS
				15	31.9 ± 3.4	CON					
Free weights	Chen et al., 2017	65–75	M	15	28.3 ± 4.4	RT	8	2	No	70%RM	BMI; BF; FFM; SMC
				15	29 ± 3.9	CON					
Free weights	Reljic et al., 2021	>18	M and F	21	37.3 ± 7.6	RT	12	2	Deficit of 500 kcal per day while maintaining proper protein consumption	Week 1–4: 50–60% 1RM; week 5–8: 60–75% 1RM; week 9–12: 70–80% 1RM	BMI; BF; SMC
				21	39.8 ± 8.9	RT 3					
				20	38.0 ± 6.3	CON					
Resistance bands	Hassannejad et al., 2017	20-50	M and F	20	42.9 ± 3.9	RT	12	3	600–800 cal 1–4 weeks, 800–1000 cal weeks 4–8, and 1000–1200 cal weeks 8–12	12–14BS	BMI; FFM; SMC; MS
				20	46.6 ± 6.9	CON					
Resistance bands	Fritz et al., 2018	60-85	F	22	27.44 ± 1.05	RT(TEB)	8	2	No	10RM	BF; FFM
				21	28.98 ± 0.86	RT(ET)					
				20	26.52 ± 0.85	CON					
Resistance bands	Huang et al., 2017	>60	F	18	27.31 ± 3.74	RT	12	3	NO	RPE 13	BMI; BF
				17	28.96 ± 3.49	CON					
Resistance bands	Liao et al., 2017	60–80	F	25	27.32 ± 3.33	RT	12	3	No	RPE 13	BF; FFM; SMC; MS
				21	28.19 ± 3.27	CON					

*BS, borg scale; RPE, rating of perceived exertion; F, female; M, male; Y, yes; N, no; BMI, body mass index; BF, body fat; FFM, fat-free mass; SMC, skeletal muscle content; MS, muscle strength; ET, elastic tube; TEB, traditional elastic band*.

### Risk of Bias of Included Studies

**Figure 2:**
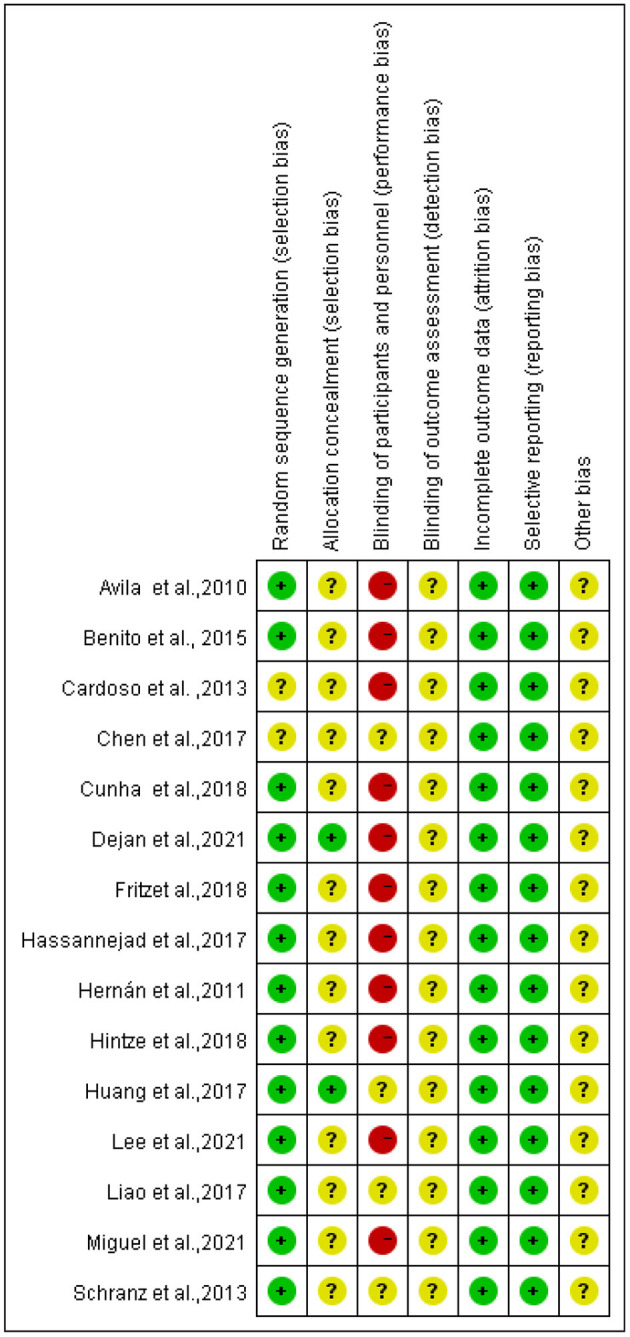
Risk of bias summary of included studies.

**Figure 3:**
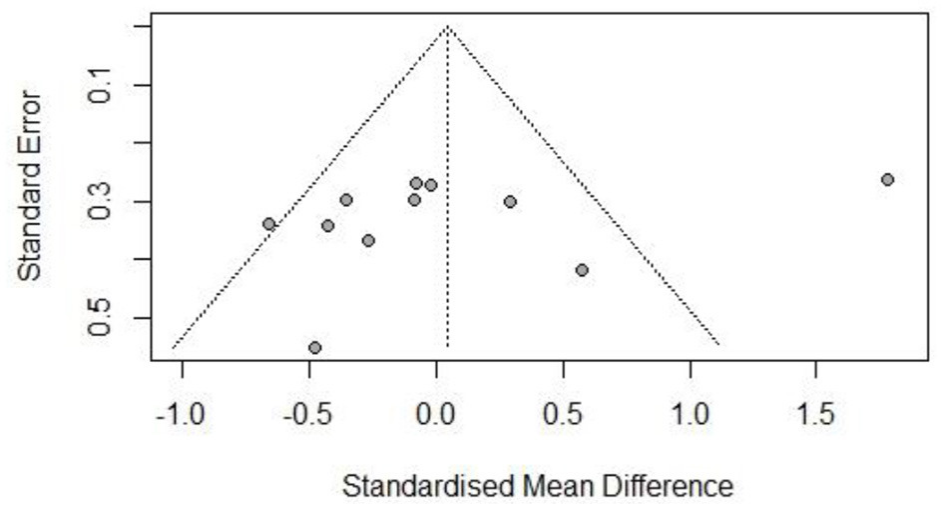
Funnel plot of body mass index (BMI).

**Figure 4:**
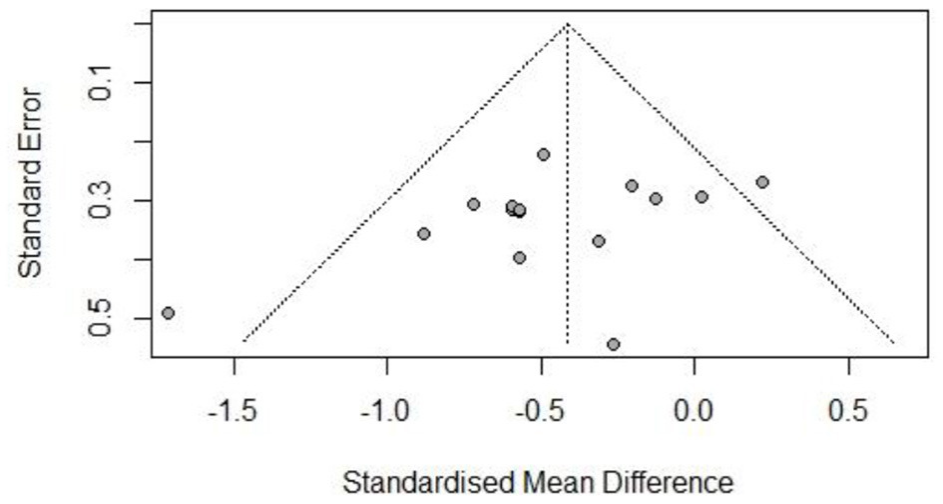
Funnel plot of body fat (BF).

**Figure 5:**
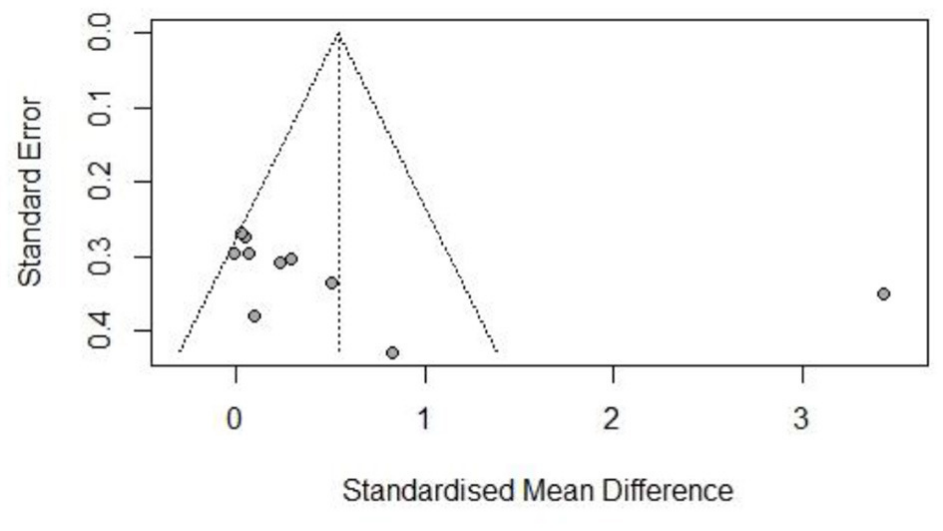
Funnel plot of free-fat mass (FFM).

The risk of bias was assessed for each included study based on Higgins and Green's study (Figure 2). A total of 15 articles were included in this study, all of which used randomization methods, with six articles mentioning the specific method of random assignment. Some articles did not report any information about allocation concealment. None of the trials met the requirement of participant blinding. However, the use of blinding did not appear to be feasible considering the exercise intervention. All articles presented full outcome indicators and stated the likelihood of reviewing the results and outcomes. All studies were unsure of the presence of other biases. Figures 3–5 show funnel plots for BMI, BF, and FFM, showing the MD of each study with its precision (SE). Visual inspection of the funnel plots revealed no significant asymmetries. Statistical tests for publication bias using the Egger regression did not reach statistical significance (Egger test for BMI, *p* = 0.28; Egger test for BF, *p* = 0.09; Egger test for FFM, *p* = 0.21).

### Effect of Intervention

#### Body Composition

**Figure 6:**
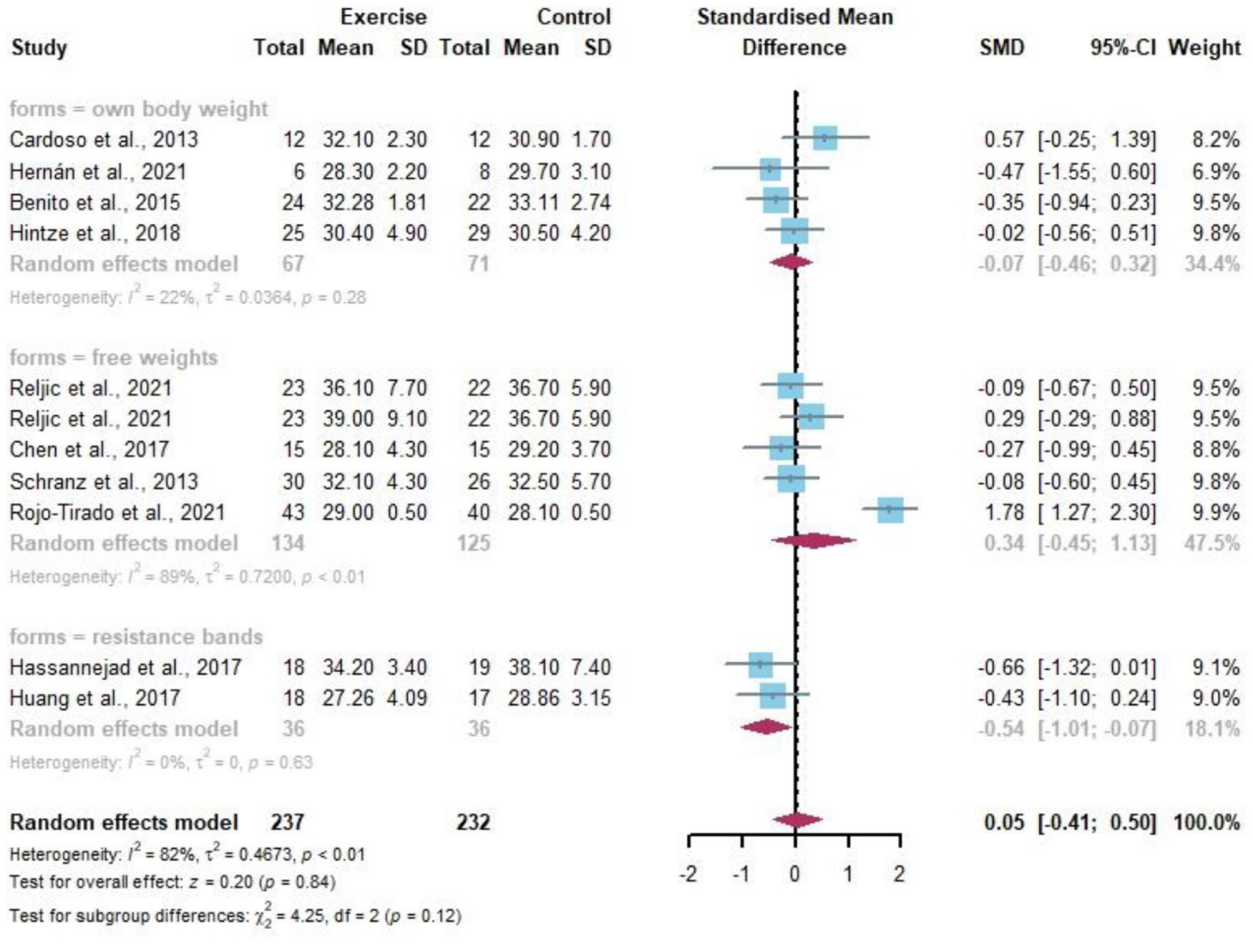
Meta-analysis of effects of different resistance exercise forms on body mass index (BMI) of overweight and/or obese individuals.

Body composition was assessed by the following four main indicators (BMI, BF, FFM, and SMC). A total of 11 data points report the change in BMI by resistance exercise (Figure 6). Random-effects models showed no statistically significant differences in BMI in the obese or/and overweight population compared to controls before and after the resistance exercise intervention (*I*^2^ = 82%, *p* = 0.84). After analyzing the different resistance types by the subgroup analysis, resistance bands well improved the BMI values of overweight and obese people (SMD −0.54, 95% CI −1.01 to −0.07, *I*^2^ = 0%), although there was no significant difference between groups.

**Figure 7:**
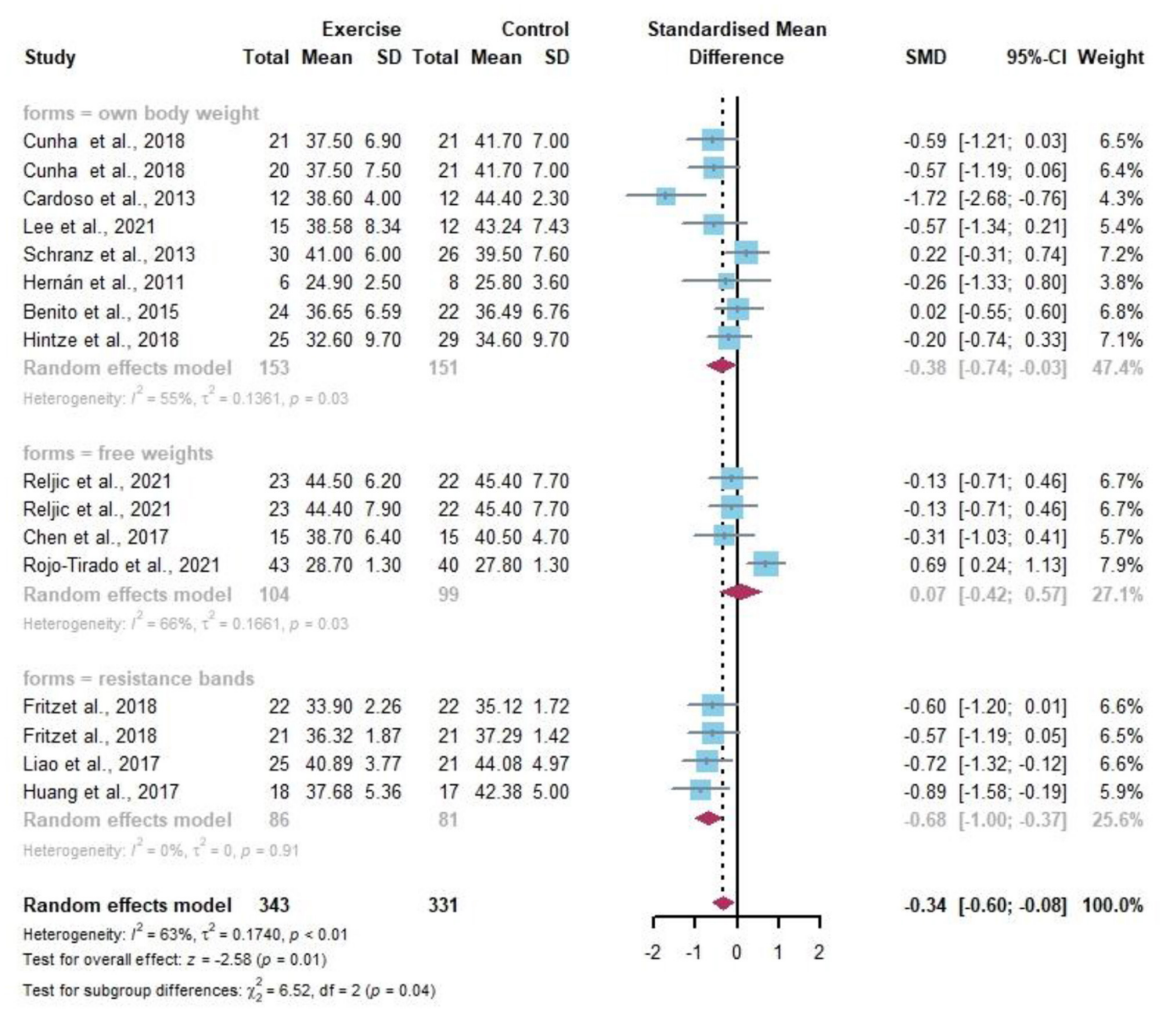
Meta-analysis of effects of different resistance exercise forms on body fat (BF) of overweight and/or obese individuals.

Sixteen data points reported changes in resistance exercise on BF (Figure 7). Random-effects models showed statistically significant differences in BF in the obese or/and overweight population before and after the resistance exercise intervention compared to the control group (*I*^2^ = 63%, *p* < 0.01). After analyzing the different resistance types by the subgroup analysis, resistance bands well improved the percentage of BF in the overweight and obese population (SMD −0.68, 95% CI −1.00 to −0.37, *I*^2^ = 0%).

**Figure 8:**
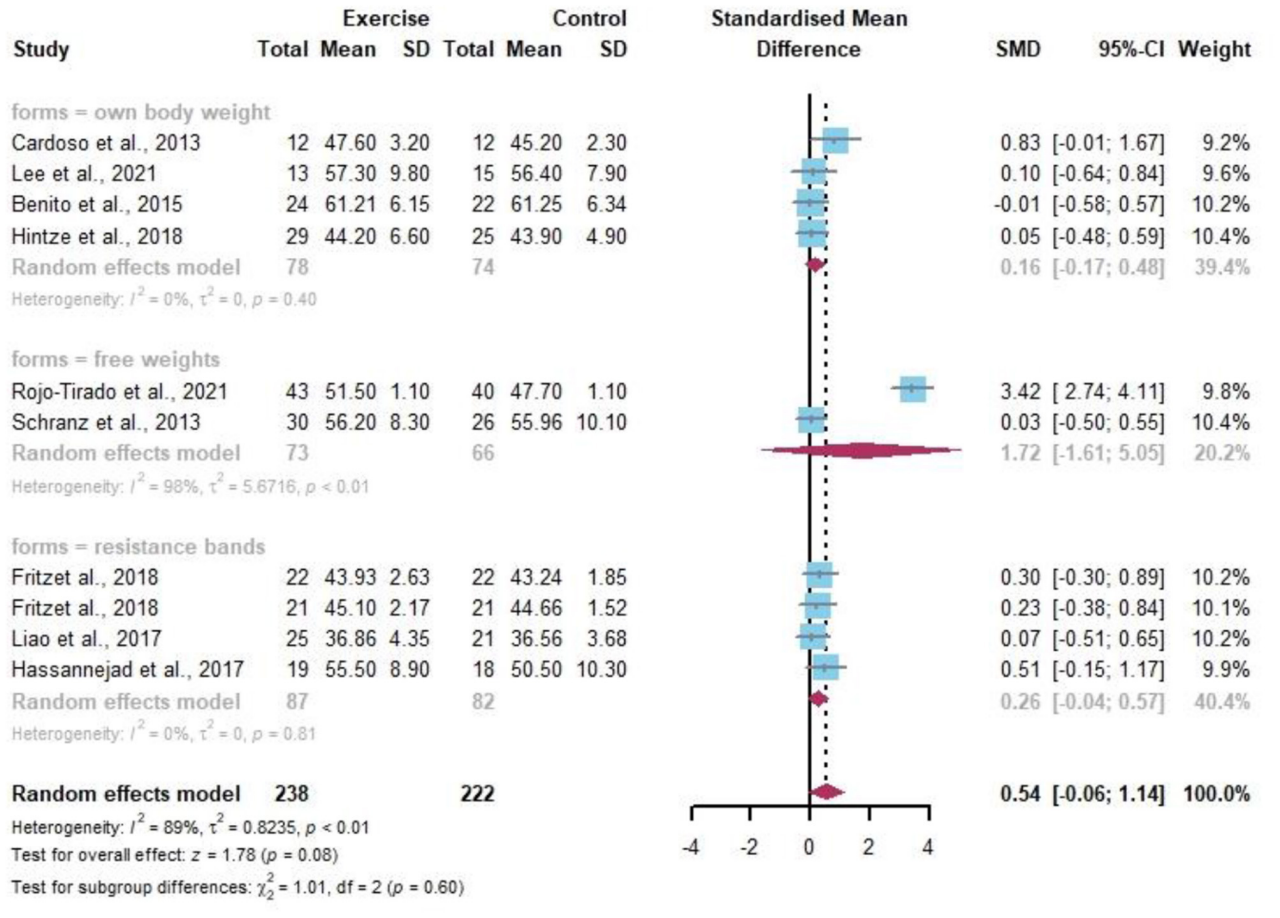
Meta-analysis of effects of different resistance exercise forms on FFM of overweight and/or obese individuals.

Ten data points reported changes in resistance exercise for FFM (Figure 8). Random-effects models showed no statistically significant differences in BMI in obese or/and overweight individuals before and after the resistance exercise intervention compared to controls (*I*^2^ = 89%, *p* = 0.06). After analysis of the different resistance types by the subgroup analysis, none of them were significantly different.

**Figure 9:**
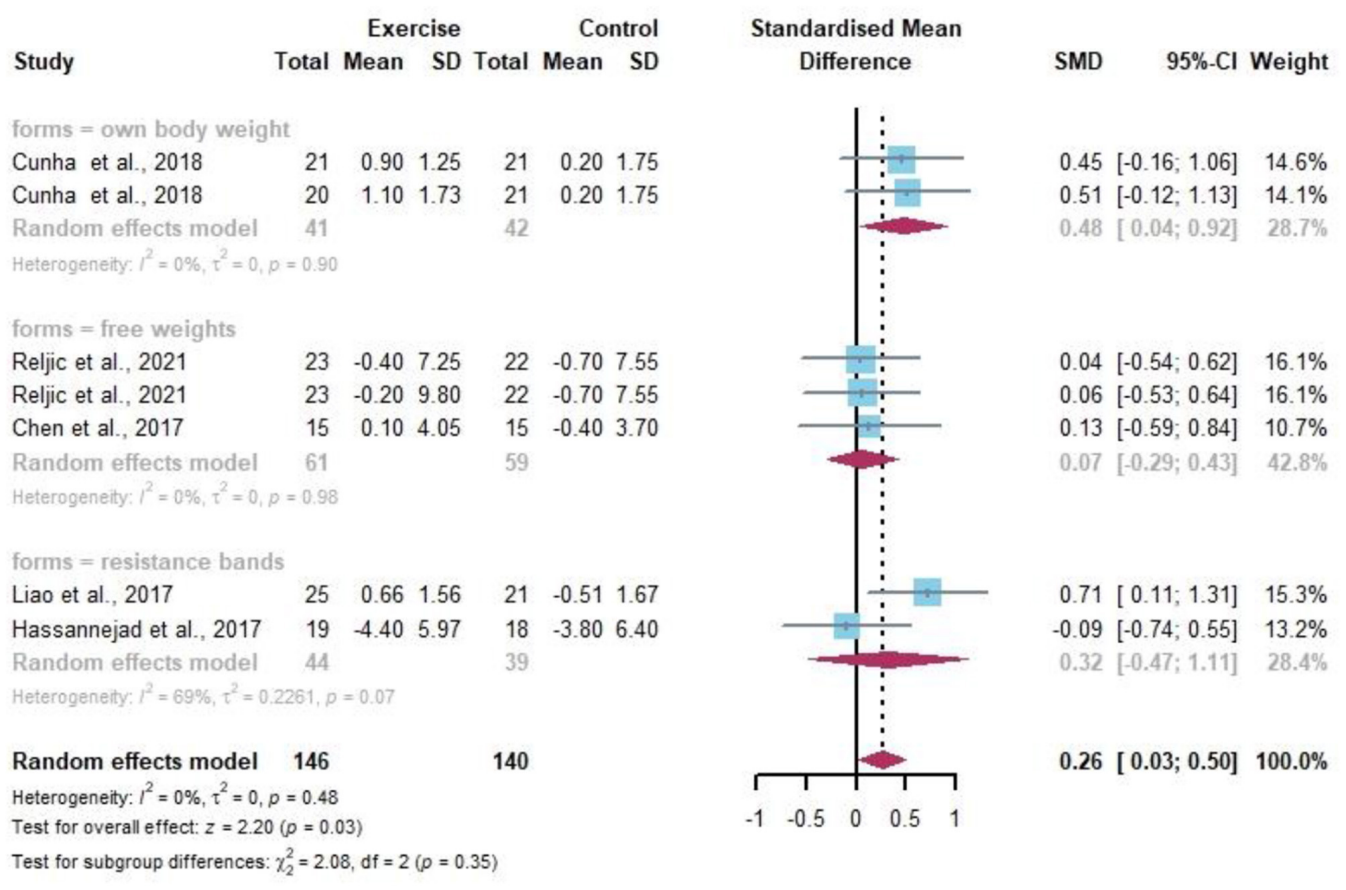
Meta-analysis of effects of different resistance exercise forms on SMC of overweight and/or obese individuals.

Seven data points reported the change in SMC by resistance exercise (Figure 9). Random-effects models showed differences and statistical significance in SMC in obese or/and overweight individuals compared to controls before and after the resistance exercise intervention (*I*^2^ = 0%, *p* = 0.03). The percentage of SMC in overweight and/or obese individuals was well improved by the subgroup analysis after different forms of resistance by own body weight (SMD 0.48, 95% CI 0.04–0.92, *I*^2^ = 0%).

### Muscle Strength

**Figure 10:**
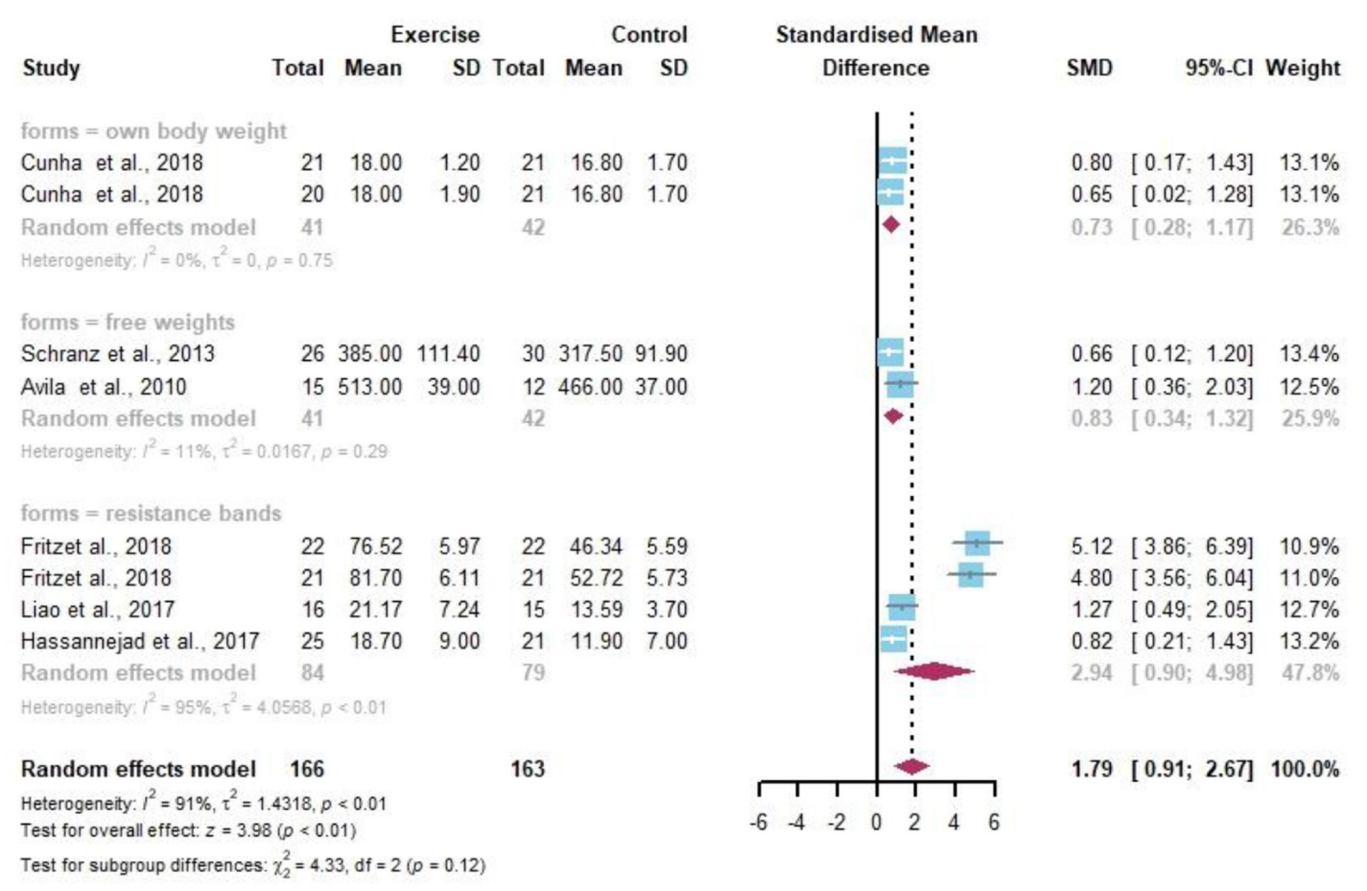
Meta-analysis of effects of different resistance exercise forms on MS of overweight and/or obese individuals.

Eight data points reported the change in resistance exercise on MS (Figure 10). The random-effects model showed a statistically significant difference in MS in the obese or/and overweight population before and after the resistance exercise intervention compared to the control group (*I*^2^ = 91%, *p* < 0.01). Sensitivity analysis showed that the effect of either article exclusion on the overall heterogeneity was not significant, indicating more stable results with high confidence. Although all three modalities were effective in increasing muscle strength, no significant differences were found between groups by the subgroup analysis.

## Discussion

This review pooled RCTs, 15 studies, and 18 trials with a total of 669 participants in a meta-analysis to assess the effects of different forms of resistance exercise on improving body composition and strength in overweight and/or obese people. By comparing different forms of resistance exercise, we found that resistance bands have the best effect on fat loss for overweight and obese people and a better effect on muscle gain for their own body weight. In addition, own body weight had the best effect on increasing muscle strength in overweight and/or obese people.

### Body Composition

Body composition is related to various physiological and pathological states, and the determination of body composition usually includes multiple indicators (Müller et al., 2012), and the evaluation of obesity should not be determined by BMI values alone but should include BF, FFM, and SMC (Bosy-Westphal and Müller, 2021), thus, its more accurate and comprehensive evaluation of its body composition. This review showed that the effect of resistance training on their BMI values and FFM was not significant, which is generally consistent with previous studies (Schranz et al., 2013). We found by the subgroup analysis of resistance exercise with different forms that own body weight and free weights did not significantly improve BMI values, BF percentage, and FFM, and free weights and resistance bands did not significantly improve SMC.

In contrast to previous studies, we found that resistance bands significantly improved BMI, although there was no significant difference between groups, and for overweight and obese people, resistance bands significantly reduced BF in overweight and obese people, and resistance bands can significantly reduce the BF value to achieve fat loss. This may be due to the special structure of resistance bands; the tensile force will gradually increase with its elongation, so the intensity of resistance training is not high, and the body mainly uses the aerobic oxidation of sugar and fat for energy supply, which can be very high to achieve the purpose of fat loss (Astorino, 2000; Stisen et al., 2006). And compared with other forms of resistance exercise, resistance bands can change the direction of resistance at will, the user can basically train most of the muscles of the body in any position and posture, so it can be very good to achieve the purpose of fat burning. But because of its resistance training intensity is not high, can not cause further stimulation of the muscle, so the effect of resistance bands on muscle gain is not ideal.

Furthermore, own body weight, as the only form of resistance exercise, can significantly increase muscle mass. For overweight and obese people, they are heavier, so the intensity of resistance bands is sufficient to stimulate the muscles and promote the thickening of muscle fibers. In addition, self-weight training can simultaneously allow our muscles to participate in the movement; rather than isolated muscle stimulation, this training is more comprehensive to increase all muscle groups, thus achieving the purpose of muscle building.

This is a very exciting finding, as it has been previously reported that obesity reduces the effect of resistance exercise on improving body composition. For example, a 6-month period of resistance exercise showed that resistance exercise was good for increasing strength but not statistically significant for improving body composition (Schranz et al., 2014). In a subsequent study, it was also found that resistance exercise for 1 year did not improve body mass in obese people (Hintze et al., 2018). So, the findings of this study certainly break with the traditional aerobic exercise that has been the main means of weight loss (Fogelholm et al., 2000; Villanova et al., 2006), especially for the overweight and obese population. After all, compared to aerobic exercise, resistance exercise can increase lean body mass and muscle mass in addition to reducing fat content (García-Hermoso et al., 2018), thus better-improving body composition. Therefore, this finding undoubtedly provides strong support for resistance training as an effective way to combat obesity, lose fat, and gain muscle.

Since caloric restriction can improve body composition by accelerated loss of fat mass and blunted increase in FFM, it has been considered effective for weight loss in overweight and obese populations in previous studies (Astrup et al., 2004). In this study, we performed the subgroup analysis with and without dietary intervention to better assess the effect of different forms of resistance exercise on body composition in overweight and obese people −1.16 to −0.07, *p* = 0.08), and FFM (SMD 0.94, 95% CI −0.33 to 2.21, *p* = 0.65) were not significantly improved. This reinforces the reference value of the differences in the effects of different forms of resistance exercise on body composition in overweight and obese people.

### Muscle Strength

Muscle strength is one of the indicators that respond to a person's functional capacity (Barbat-Artigas et al., 2014). Lower muscle strength leads to reduced physical mobility and increased risk of falls and fractures, especially in the aging population (Visser et al., 2005; Newman et al., 2006). The muscle strengths incorporated in this study are all absolute strengths, which are particularly important for grasping the intensity of exercise and reducing the occurrence of sports injuries. There should be no doubt that resistance exercise increases muscle mass (Peterson and Gordon, 2011; Candow et al., 2012) and muscle strength (Peterson et al., 2010; Larsson et al., 2015), and this is equally supported by the literature on obese and overweight population (Avila et al., 2010; Cunha et al., 2018). Current studies have mainly shown that resistance exercise increases muscle mass and strength by increasing the expression of relevant skeletal muscle synthesis proteins and decreasing catabolic levels (Dreyer et al., 2010; Peterson et al., 2010; Agergaard et al., 2017).

In this study, by comparing the three forms of resistance exercises, it was found that there was no significant difference between the three exercise forms for strength improvement, but the resistance bands showed a better trend for muscle strength improvement compared to the other two forms, and the subgroup analysis showed low heterogeneity, which can also better indicate that the data are more stable and reliable. The reliability of the data was high. Due to the specific structure of resistance bands, the tensile force increases gradually with their elongation, leading to changes in the muscle itself (muscle fiber type, muscle structure, myofilament density, structure of connective tissue, and tendons) and an increase in muscle nerve adaptation. In particular, the results of this article show that the magnitude of the effect of resistance exercise on MS appears to be greater than that on SMC in overweight or obese people, implying that the effect of resistance exercise on muscle neural recruitment and/or muscle contractile function is greater than the effect on “fiber thickening.” Therefore, for overweight and obese people, there may be a decrease in muscle anabolism, and the mechanism for increasing muscle strength is likely to be mainly resistance exercise that increases muscle neural recruitment and/or muscle contractile function. Compared to own body weight and free weights, the intensity of resistance training can be customized and optimized, and it is more functional, usually with the coordination of multiple muscle groups, so it is more effective in increasing muscle strength. Additionally, because overweight and obese people are heavier, the pressure on the joints is greater than that of healthy people, so resistance training with free weights and own body weight will undoubtedly increase the pressure on the joints, which will easily cause damage to the joints and is not suitable for overweight and obese people. In addition, resistance bands, unlike other resistance training forms, are not controlled by the training site and are very light to practice, so they will greatly benefit the health of all people.

### Strength and Limitations

This review is the first to examine the effects of different forms of resistance exercise on body composition and muscle strength in overweight and/or obese populations with outcome measures including BMI, BF, FFM, SMC, and MS. In addition, the evidence from RCTs will be more rigorous and objective than that from case studies. Compared to previous studies, this study includes different forms of resistance training to evaluate which resistance training forms can better improve body composition and strength, thus making training more precise and a significant contribution to personalized exercise prescription. Additionally, this review is a meta-analysis, which improves the statistical power of the article, provides more precise data, and addresses the reasons for conflicting results between different experimental studies.

Although the studies included in this review may not be typical of resistance exercise to assess changes in body composition and strength in different forms in obese populations, they provide a benchmark. It is suggested that the effects of resistance exercise on body composition and strength are beneficial and that different forms of resistance exercise can be used to achieve different effects (fat loss, muscle gain, strength gain, etc.), especially in overweight and obese populations. In terms of overweight and obese people, choosing resistance bands for fat loss and resistance exercise for their own body weight to increase muscle mass and increase muscle strength would be the best choice. Although the subgroup analysis of the factors that may influence the results of this study (caloric restriction, exercise volume, etc.) did not significantly interfere with the results of the experiment, however, due to the small number of studies available, especially for the subgroup analysis, the included literature is more limited and single, which may be limiting. In addition, the age of the subjects in their own body weight was ≥12, the age of the subjects in the free weights was ≥13, and the age of the subjects in the resistance bands was ≥20. The subjects in the resistance bands were indeed slightly older, but overall, the age range of the subjects included in all three training modalities was wide. Due to the limitations of the included literature, the variable of age was not taken into account in this paper; therefore, future studies on different resistance exercise forms should be increased, especially for overweight and obese people for different age groups, and studies combining dietary interventions and resistance training should be increased in order to better inform future personalized exercise prescriptions.

## Conclusion

This systematic review and meta-analysis show that different forms of resistance exercise have different effects on overweight and obese individuals. Resistance bands can improve body composition by decreasing BF. Resistance bands can improve body composition by decreasing BF but are more effective at increasing muscle mass and own body weight. Therefore, for overweight and obese people, resistance bands can be used to lose fat, while resistance weights can be used to gain muscle and maintain muscle mass, thus improving body composition. However, given the limitations outlined in this study, it is necessary to take the results with a grain of salt. More RCTs with large sample sizes are needed to better understand the effectiveness of different forms of resistance exercise interventions on body composition and muscle strength.

## Data Availability Statement

The original contributions presented in the study are included in the article/supplementary material, further inquiries can be directed to the corresponding author/s.

## Author Contributions

XL and XS designed the systematic review. JLu and YG analyzed the data. QM prepared the figures and table. XL wrote the manuscript. YS and JLi revised the manuscript. All authors reviewed and approved the manuscript.

## Funding

This study was supported by the National Key Research and Development Program of China (Grant No. 2018YFC2000600) and the Fok Ying Tung Education Foundation (No. 161094).

## Conflict of Interest

The authors declare that the research was conducted in the absence of any commercial or financial relationships that could be construed as a potential conflict of interest.

## Publisher's Note

All claims expressed in this article are solely those of the authors and do not necessarily represent those of their affiliated organizations, or those of the publisher, the editors and the reviewers. Any product that may be evaluated in this article, or claim that may be made by its manufacturer, is not guaranteed or endorsed by the publisher.
